# Prevalence and Risk Factors of Suspected Glaucoma Identified Through a Point-of-Care Device in the Eye Camps of Rural Shivamogga, Karnataka

**DOI:** 10.7759/cureus.97113

**Published:** 2025-11-17

**Authors:** Anupama K, Balu PS, Gayathri Mahadevan, Swathi HJ

**Affiliations:** 1 Community Medicine, Subbaiah Institute of Medical Sciences, Shivamogga, IND; 2 Centre for AI Research and E-Health, Subbaiah Research Institute, Shivamogga, IND; 3 Ophthalmology, Subbaiah Institute of Medical Sciences, Shivamogga, IND

**Keywords:** point of care, rural area, screening camp, shivamogga, suspected glaucoma

## Abstract

Background: Glaucoma, a progressive optic neuropathy and leading cause of irreversible blindness worldwide, is frequently asymptomatic until advanced stages, making early detection crucial to prevent vision loss. Conventional diagnosis is complex, requiring multiple examinations and specialist expertise. Digital tools like portable tonometry offer promising alternatives for community-based screening.

Objectives: To screen adult patients (≥40 years) attending District Blindness Camp Shivamogga (DBCS) screening camps for glaucoma using a point-of-care device. To assess the prevalence of suspected glaucoma in the screened population. To assess the risk factors associated with suspected glaucoma.

Methods: This community-based cross-sectional study, conducted over a six-month period, recruited 904 adults ≥40 years using simple random sampling in the DBCS eye camp. After obtaining consent, demographic data, comorbidities, and ocular history were collected, followed by intraocular pressure (IOP) measurement using a point-of-care rebound tonometer. Participants flagged as glaucoma suspects were referred to a tertiary center for confirmatory evaluation.

Results: A total of 904 adults ≥40 years were screened, with most aged 41-50 years (31.4%) and a female predominance (64.1%). Diabetes (30.7%) and hypertension (18.3%) were common. Elevated IOP was detected in 2.4% of right and 2.4% of left eyes, and these glaucoma suspects were referred for confirmatory evaluation. Hypertension and diabetes mellitus were found to have a statistically significant association with suspected glaucoma (p<0.001).

Conclusion: This study demonstrates the feasibility of incorporating point-of-care tonometry into community eye camps for early glaucoma detection. By combining portability, automated analysis, and referral linkage, this model can address the high proportion of undiagnosed glaucoma in resource-limited settings and reduce the burden of preventable blindness.

## Introduction

Glaucoma, a progressive optic neuropathy, is the leading cause of irreversible blindness worldwide and is usually asymptomatic until its advanced stages [1]. This highlights the importance of promptly detecting and effectively managing glaucoma to prevent its impact on an individual's quality of life. In 2013, the worldwide prevalence of glaucoma was estimated at 64.3 million people [2]. This number increased to 76.0 million by 2020, with projections anticipating a rise to about 111.8 million by 2040 [2]. Glaucoma is marked by structural alterations affecting the optic nerve head (ONH) and the retinal nerve fiber layer (RNFL) [1]. In developing countries, 90% of people are unaware they have glaucoma [3]. This is critical because patients are generally asymptomatic in the early stages until central vision is affected in the advanced stages. Vision-related quality of life (VRQOL) in glaucoma is influenced by factors such as visual function level, education, income, number of glaucoma medications, follow-up duration, and other variables [4]. As glaucoma severity increases, VRQOL typically declines, with advanced stages showing poorer outcomes than mild or moderate glaucoma [4,5]. Glaucoma detection is complex, subjective, and time-consuming, requiring multiple examinations and clinical expertise. Imaging techniques play a key role in assessing structural abnormalities. Monoscopic fundus photography has shown similar diagnostic accuracy for glaucoma detection compared with stereoscopic photography [6,7]. Thus, digital automated device-based screening for glaucomatous changes is practically beneficial. This study aims to screen adult patients (aged ≥40 years) attending an eye camp for glaucoma using a point-of-care device [8] and to assess the prevalence of suspected glaucoma and associated risk factors in the screened population.

## Materials and methods

This was a community-based cross-sectional study conducted over six months, from January 2025 to July 2025. The study was conducted at eye screening camps organized by the District Blindness Control Society (DBCS) in Shivamogga District, Karnataka, India. The study population included adult participants aged 40 years and above who visited the DBCS eye screening camps during the study. A universal sampling technique was used. All eligible individuals aged 40 years and above who attended the DBCS eye screening camps during the study period and met the inclusion criteria were enrolled.

Inclusion criteria

The study included adults aged 40 years and above who were willing to participate and provided written informed consent.

Exclusion criteria

Bedridden or terminally ill individuals and those with a history of complete vision loss in both eyes were excluded from the study.

Methodology

Following approval from the Institutional Ethics Committee, the study was carried out in district-based eye screening camps organized by the DBCS in rural areas of Shivamogga District. Written informed consent was obtained from all participants before inclusion in the study.

Data collection

Detailed demographic information, such as age, gender, family history of glaucoma, and systemic conditions like diabetes mellitus, hypertension, or myopia, was collected from all participants. Relevant medical and ocular symptoms were recorded on a structured proforma developed through Google Forms for standardized documentation and ease of analysis.

Screening procedure

The screening began with visual acuity measurement using a Snellen chart. Intraocular pressure (IOP) was measured for both eyes with a point-of-care tonometer, a handheld device that automatically calculated IOP values and flagged values above the normal range. For this study, an IOP of ≤21 mmHg was considered normal, while levels above 21 mmHg were classified as glaucoma suspects. For accuracy, any patient with an IOP above 21 mmHg was remeasured, and the mean of two readings was taken as the final. Participants were then categorized into two groups: those with normal IOP and glaucoma suspects. Patients identified as glaucoma suspects were referred to a tertiary eye center for confirmatory testing, including gonioscopy, field testing, and optical coherence tomography (OCT) as appropriate.

Principle of rebound tonometry

The point-of-care tonometer used in this study employs rebound technology, which differs from conventional air-puff or Goldmann applanation tonometry. A light probe is propelled toward the cornea by an electromagnetic field. After briefly contacting the cornea, the probe rebounds, and the device records its deceleration and contact time. These parameters are proportional to IOP: higher IOP values produce a quicker rebound and shorter contact time, while lower IOP values result in a slower rebound and longer contact time. This technique is noninvasive, quick, and well-tolerated. It also does not require topical anesthetic drops, making it especially useful for field-based screening and for patients who are uncooperative with conventional tonometry. Thus, rebound tonometry provides a valid, effective, and patient-friendly method for mass ocular pressure measurement in field settings.

Statistical analysis

Data were entered in Microsoft Excel (Microsoft Corp., Redmond, WA) and analyzed using SPSS version 20 (IBM Inc., Armonk, New York). Descriptive statistics summarized demographic and clinical variables; continuous data were presented as mean±SD, while categorical data were expressed as frequencies and percentages. Associations between risk factors and glaucoma were evaluated using the chi-square test for categorical variables and the t-test for continuous variables. A p-value <0.05 was considered statistically significant.

## Results

Table 1 shows the sociodemographic details of the patients. Most were aged between 41 and 50 years (293 patients, 32.4%), followed by those aged 51 to 60 years (283 patients, 31.3%), 61 to 70 years (243 patients, 26.9%), 71 to 80 years (73 patients, 8.1%), and those over 80 years (12 patients, 1.3%). Of the total 904 patients, most were female (580, 64.1%), while male patients made up 324 (35.9%). Regarding comorbidities, 163 patients (18.03%) had diabetes mellitus, and 272 patients (30.07%) had hypertension.

**Table 1:** Sociodemographic details of the study participants HTN, hypertension; DM, diabetes mellitus

Sociodemographic details	Sub-variables	Frequency	Percentage
Age	41-50	293	32.4%
	51-60	283	31.3%
	61-70	243	26.9%
	71-80	73	8.1%
	>80	12	1.3%
Gender	Male	324	35.9%
	Female	580	64.1%
Comorbidities	HTN	272	30.07%
	DM	163	18.03%
Total		904	100%

**Figure 1:**
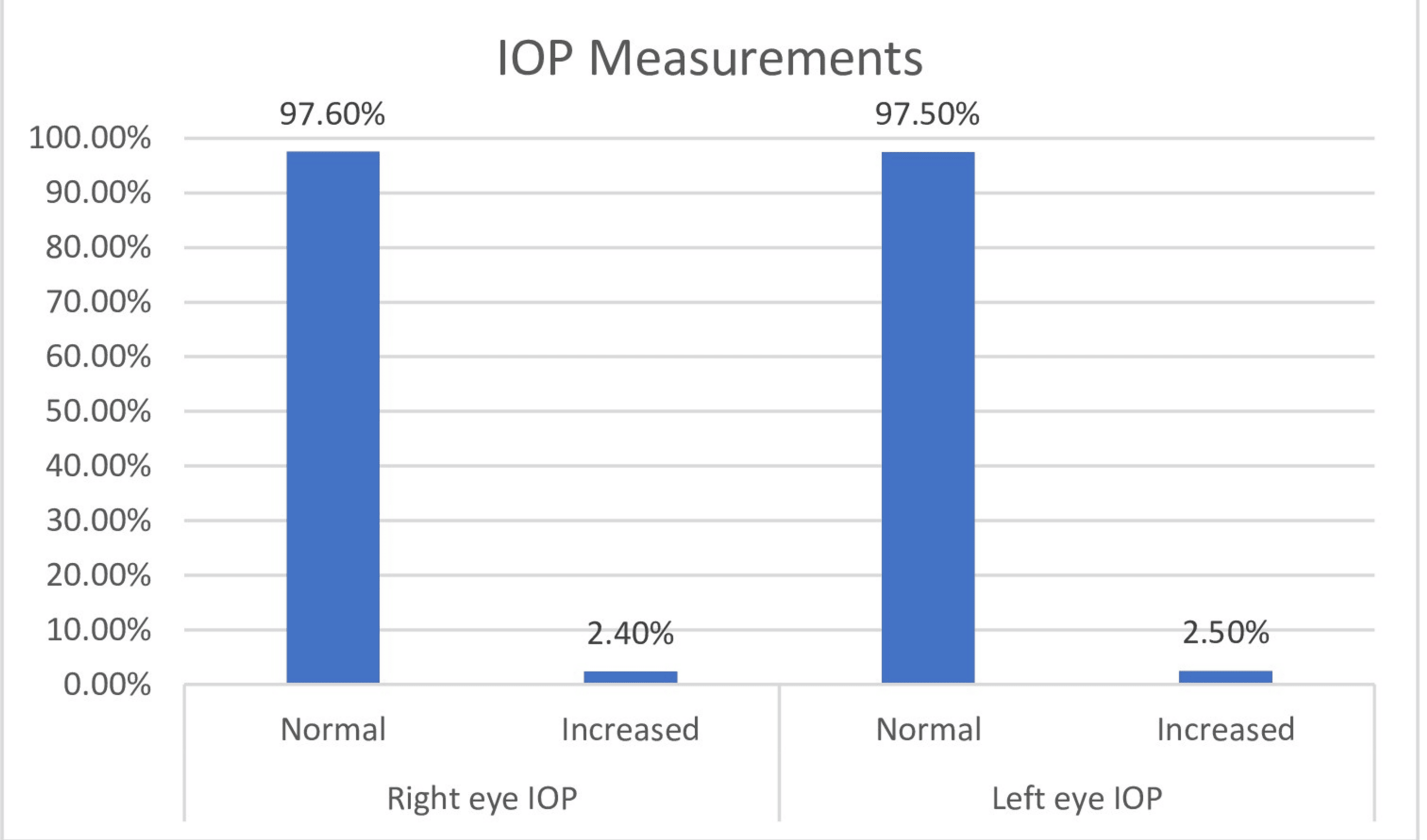
IOP measurement of the study participants IOP, intraocular pressure

Table 2 shows the IOP measurements. Out of 904 patients, 22 (2.4%) were found to have increased IOP in the right eye, and 23 (2.5%) were found to have increased IOP in the left eye. The combined increased IOP among the study participants was 26 (2.8%). Figure 1 shows the IOP measurements of the study participants.

**Table 2:** IOP measurement of the study participants IOP, intraocular pressure

IOP	Sub-variables	Frequency	Percentage
Right eye IOP	Normal	882	97.6%
	Increased	22	2.4%
Left eye IOP	Normal	881	97.5%
	Increased	23	2.5%
Total		904	100%

Table 3 shows the association between risk factors and suspected glaucoma. Age and gender had no association with suspected glaucoma. Of the 26 patients with elevated IOP, 24 had hypertension (92.3%) and 19 had diabetes mellitus (73.1%). The prevalence in hypertensive patients was 24/272 (8.8%), and the prevalence in diabetic patients was 19/163 (11.7%). Hence, hypertension and diabetes mellitus were found to have a statistically significant association with suspected glaucoma (p<0.001).

**Table 3:** Association between risk factors and suspected glaucoma (increased IOP) IOP, intraocular pressure

Age	Normal	Increased IOP	Total	P-value
41-50	283 (32.2%)	10 (38.5%)	293 (32.4%)	P=0.85
51-60	274 (31.2%)	9 (34.6%)	283 (31.3%)	
61-70	238 (27.1%)	5 (19.2%)	243 (100%)	
71-80	71 (8.1%)	2 (7.7%)	73 (100%)	
>80	12 (1.4%)	0	12 (100%)	
Gender				
Male	313 (96.6%)	11 (42.3%)	324 (35.8%)	P=0.485
Female	565 (64.4%)	15 (57.7%)	580 (64.2%)	
Hypertension				
Present	248 (28.2%)	24 (92.3%)	272 (30.1%)	P<0.001
Absent	628 (70.8%)	2 (7.7%)	630 (69.0%)	
Diabetes mellitus				
Present	144 (16.4%)	19 (73.1%)	163 (18.14%)	P<0.001
Absent	732 (82.7%)	7 (26.9%)	741 (81.96%)	
Total	878 (100%)	26 (100%)	904 (100%)	

## Discussion

In our present suspected glaucoma screening study (n=904), most participants were aged 41-70 years (90.6%), with the highest percentage in the 41-50 years group (32.4%). A predominance of females (64.1%) was observed, consistent with reports that women are more likely to seek community health programs owing to health-seeking behavior. Comorbidities were prevalent, with diabetes mellitus (18.14%) and hypertension (30.07%), in keeping with systemic vascular risks commonly seen with ocular morbidity. Increased IOP was noted in 2.5% of right eyes and 2.4% of left eyes, indicating the importance of tonometry in detecting glaucoma suspects in outreach services.

In contrast with Senthil et al. (n=213; mean age 59±15 years), who had 60.5% confirmed glaucoma, 15.5% disc suspects, and 24% non-glaucoma [9], our study had fewer suspected glaucoma cases. This discrepancy stems from the study setting: their hospital-based cohort was enriched with symptomatic or high-risk individuals, whereas our community-based camp comprised a larger, frequently asymptomatic population.

The Chennai Glaucoma Study (CGS) also found a prevalence of 3.51% in urban and 1.62% in rural populations, with >90% undiagnosed [10]. This aligns with our results, where raised IOP may indicate an undiagnosed disease. The Andhra Pradesh Eye Disease Study also found greater prevalence of angle-closure glaucoma in women and older age groups [11], consistent with our female-dominant group. Together, these studies validate community screening as a practical and fair method of early glaucoma detection in India.

In the present community-based study, there was a strong association between systemic comorbidities and the risk of suspected glaucoma, with both diabetes mellitus and hypertension strongly correlating with high IOP. Of the 26 patients with elevated IOP, 24 had hypertension (92.3%) and 19 had diabetes mellitus (73.1%). The prevalence in hypertensive patients was 24/272 (8.8%), and the prevalence in diabetic patients was 19/163 (11.7%), suggesting that these systemic conditions may be significant in the pathogenesis of glaucoma. However, gender and age were not found to be significantly associated with suspected glaucoma in this population.

These results are consistent with worldwide studies. The Blue Mountains Eye Study in Australia demonstrated that systemic hypertension was an independent risk factor for primary open-angle glaucoma, supporting the vascular hypothesis in the pathogenesis of glaucoma [12]. Likewise, in the Barbados Eye Study, hypertension and diabetes were significantly related to increased IOP and higher glaucoma prevalence [13]. Klein et al. also reported increased IOP in diabetic individuals and postulated a role for metabolic dysfunction in modifying aqueous humor dynamics and optic nerve susceptibility [14].

Indian studies also support our findings. The CGS documented that diabetes was directly linked with elevated IOP and glaucoma risk, especially in urban populations where metabolic stress may be worsened by lifestyle factors [10]. Similarly, the Vellore Eye Study emphasized that hypertension increased glaucoma risk, potentially through compromised autoregulation of ONH perfusion [15]. These similarities indicate that systemic comorbidities are common risk factors across populations.

Limitations

The study had limited diagnostic confirmation. Elevated IOP and suspicion of glaucoma were identified using the point-of-care tool [8], but confirmatory tests such as gonioscopy, visual field testing, or optic nerve head imaging (OCT) were not performed in the camp setting. Geographic and demographic constraints were also present, as the study was conducted in a specific region and included mostly participants aged ≥40 years, so the results may not reflect younger age groups or other populations.

## Conclusions

This study emphasizes the viability of glaucoma screening in community eye camps employing the point-of-care tool. The majority of participants were middle-aged or older, with a female preponderance, representing the target population that can benefit most from outreach services. The high prevalence of hypertension and diabetes underscores the benefit of integrated screening. Although raised IOP was found in a minority, these individuals represent undiagnosed glaucoma suspects. Portable tonometry assessment presents an effective, scalable approach to improving early identification and referrals.

It is recommended to use digital point-of-care tonometry for glaucoma screening, as these portable, automated devices facilitate easy field assessment, especially among adults aged ≥40 years and those with coexisting diabetes and hypertension. However, the higher price of such devices compared with traditional tonometers may limit broader adaptation. Capacity building of the primary health workforce, establishing an effective referral pathway, and scaling up this model at the national level can increase early detection, reduce the disease burden associated with undiagnosed glaucoma, and prevent unnecessary blindness.
